# Mass Spectrometry of RNA-Binding Proteins during Liquid–Liquid Phase Separation Reveals Distinct Assembly Mechanisms and Droplet Architectures

**DOI:** 10.1021/jacs.3c00932

**Published:** 2023-05-05

**Authors:** Cagla Sahin, Aikaterini Motso, Xinyu Gu, Hannes Feyrer, Dilraj Lama, Tina Arndt, Anna Rising, Genis Valentin Gese, B. Martin Hällberg, Erik. G. Marklund, Nicholas P. Schafer, Katja Petzold, Kaare Teilum, Peter G. Wolynes, Michael Landreh

**Affiliations:** † Department of Microbiology, Tumor and Cell Biology, Karolinska Institutet − Biomedicum, Solnavägen 9, 17165 Solna, Sweden; ‡ Structural Biology and NMR Laboratory and the Linderstrøm-Lang Centre for Protein Science, Department of Biology, University of Copenhagen, Ole Maaløes vej 5, 2200 Copenhagen, Denmark; § Center for Theoretical Biological Physics, Rice University, Houston, Texas 77005, United States; ∥ Department of Chemistry, Rice University, Houston, Texas 77005, United States; ⊥ Department of Medical Biochemistry and Biophysics, Karolinska Institutet − Biomedicum, Solnavägen 9, 17165 Solna, Sweden; # Department of Biosciences and Nutrition, Karolinska Institutet, S-141 57 Huddinge, Sweden; ∇ Department of Anatomy, Physiology and Biochemistry, Swedish University of Agricultural Sciences, Box 7011, S-750 07 Uppsala, Sweden; ○ Department of Cell and Molecular Biology, Karolinska Institutet − Biomedicum, Solnavägen 9, 171 65 Stockholm, Sweden; ◆ Department of Chemistry - BMC, Uppsala University, Box 576, 751 23 Uppsala, Sweden; ¶ Department of Cell- and Molecular Biology, Uppsala University, Box 596, 751 24 Uppsala, Sweden; ⬢ Department of Medical Biochemistry and Microbiology, Uppsala University, 751 24 Uppsala, Sweden

## Abstract

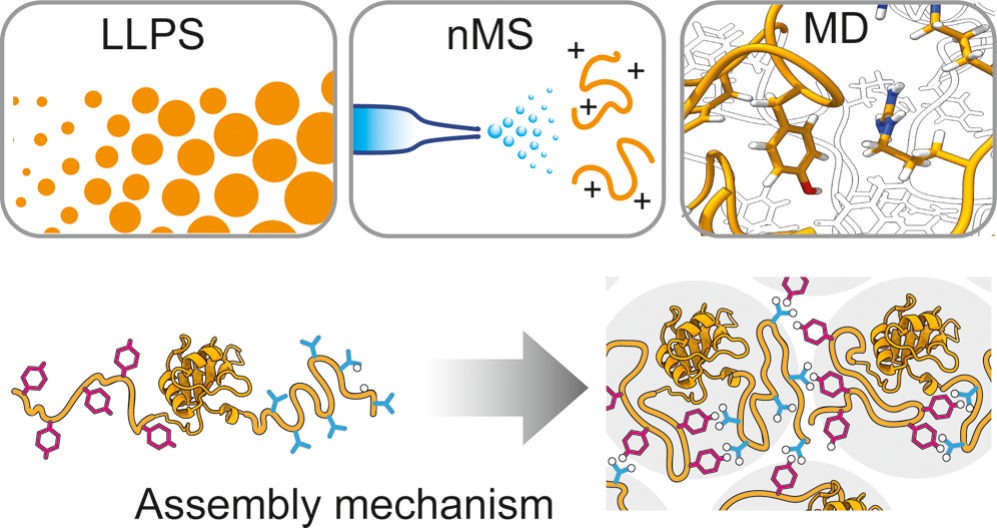

Liquid–liquid phase separation (LLPS) of heterogeneous ribonucleoproteins (hnRNPs) drives the formation of membraneless organelles, but structural information about their assembled states is still lacking. Here, we address this challenge through a combination of protein engineering, native ion mobility mass spectrometry, and molecular dynamics simulations. We used an LLPS-compatible spider silk domain and pH changes to control the self-assembly of the hnRNPs FUS, TDP-43, and hCPEB3, which are implicated in neurodegeneration, cancer, and memory storage. By releasing the proteins inside the mass spectrometer from their native assemblies, we could monitor conformational changes associated with liquid–liquid phase separation. We find that FUS monomers undergo an unfolded-to-globular transition, whereas TDP-43 oligomerizes into partially disordered dimers and trimers. hCPEB3, on the other hand, remains fully disordered with a preference for fibrillar aggregation over LLPS. The divergent assembly mechanisms revealed by ion mobility mass spectrometry of soluble protein species that exist under LLPS conditions suggest structurally distinct complexes inside liquid droplets that may impact RNA processing and translation depending on biological context.

## Introduction

Liquid–liquid phase separation (LLPS) of proteins into liquid droplets is the governing principle for the formation of membraneless organelles and controls diverse biological processes from ribosome assembly to RNA processing.^1,2^ Genome-wide analyses have revealed that the ability to form liquid droplets via LLPS is a common feature of the proteome.^3^ These findings raise the interesting possibility that LLPS is a widespread property of polypeptide chains and can even be found in globular proteins.^4,5^ The ability to form amyloid-like fibrils is similarly common across the proteome^6^ and can in turn be counteracted by LLPS.^7^ Interestingly, both LLPS and amyloid formation have been observed for heterogeneous ribonucleoproteins (hnRNPs). hnRNPs are a diverse family of proteins with a modular architecture composed of folded RNA recognition motifs (RRMs) and disordered low-complexity domains (LCDs) with prion-like properties.^8,9^ hnRNPs readily form liquid droplets *in vitro* and *in vivo*, and the biological relevance of their droplet states for the cellular RNA metabolism have been clearly established.^10^

The perhaps best-understood hnRNPs are Fused in Sarcoma (FUS) and Transactive Response DNA-binding protein 43 (TDP-43), which are components of stress granules and involved in the cellular RNA metabolism. Both proteins have a strong propensity to form fibrillar aggregates *in vitro* and *in vivo*(11−13) and have been identified as major components in neuronal inclusion bodies from patients with amyotrophic lateral sclerosis (ALS), and, for TDP-43, also in patients with limbic-age-related TDP-43 encephalopathy (LATE), which has symptoms similar to Alzheimer’s disease.^10,14,15^ It has been proposed that aberrant LLPS can give rise to toxic protein aggregates and thus become a driving factor of neurodegenerative processes.^8^ On the other hand, fibril formation has been identified as a functional feature of the human cytoplasmic polyadenylation element binding protein 3 (hCPEB3), an hnRNP that is a key regulator of synaptic plasticity and long-term memory formation.^16^ Its homologues from *Drosophila melanogaster* (Orb2), *Aplysia californica* (apCPEB), and mouse (mCPEB3) are functional prions that regulate transcriptional activity by assembling into fibril-like structures.^16^ Yet several CPEB proteins have been found to also undergo LLPS, including mCPEB3, hCPEB3, and Orb2, albeit only under specific conditions such as SUMOylation,^17^ in crowding agents,^18^ or as precursor of fibril formation.^19^

The fact that several hnRNPs can adopt multiple competing assembly states raises the question whether there are distinct conformational signatures that can be associated with LLPS. Identifying structural features that distinguish pathogenic from physiological assemblies would be an important step toward pharmacological intervention in toxic aggregation. Although the sequence requirements for LLPS are comparatively well studied,^2,4,20^ we lack an understanding of how these translate into a three-dimensional architecture. NMR spectroscopy has provided valuable information;^21^ however, obtaining and comparing structural determinants of droplet formation remains challenging due to the poor solubility and low conformational stability of many hnRNPs. For example, full-length FUS and TDP-43 require the presence of strong denaturants or fusion to expression tags, which prevent aggregation as well as LLPS, rendering purified proteins either non-native or nonfunctional.^22−25^ Furthermore, the choice of renaturation strategy can affect the balance between LLPS and aggregation, leading to conflicting observations.^26,27^

Here, we develop a pH-responsive LLPS system by fusing aggregation-prone hnRNPs to an engineered spider silk domain and use native mass spectrometry (nMS) in combination with ion mobility spectroscopy (IM) to identify conformational changes that are associated with droplet formation. Using MD simulations, we find that despite similar domain organizations, hnRNPs adopt distinct conformational states during assembly, which affect the orientation of bound RNAs and may distinguish specific biological contexts.

## Results

### Assembly of FUS and TDP-43 into Liquid Droplets Can Be Observed by nMS

**Figure 1:**
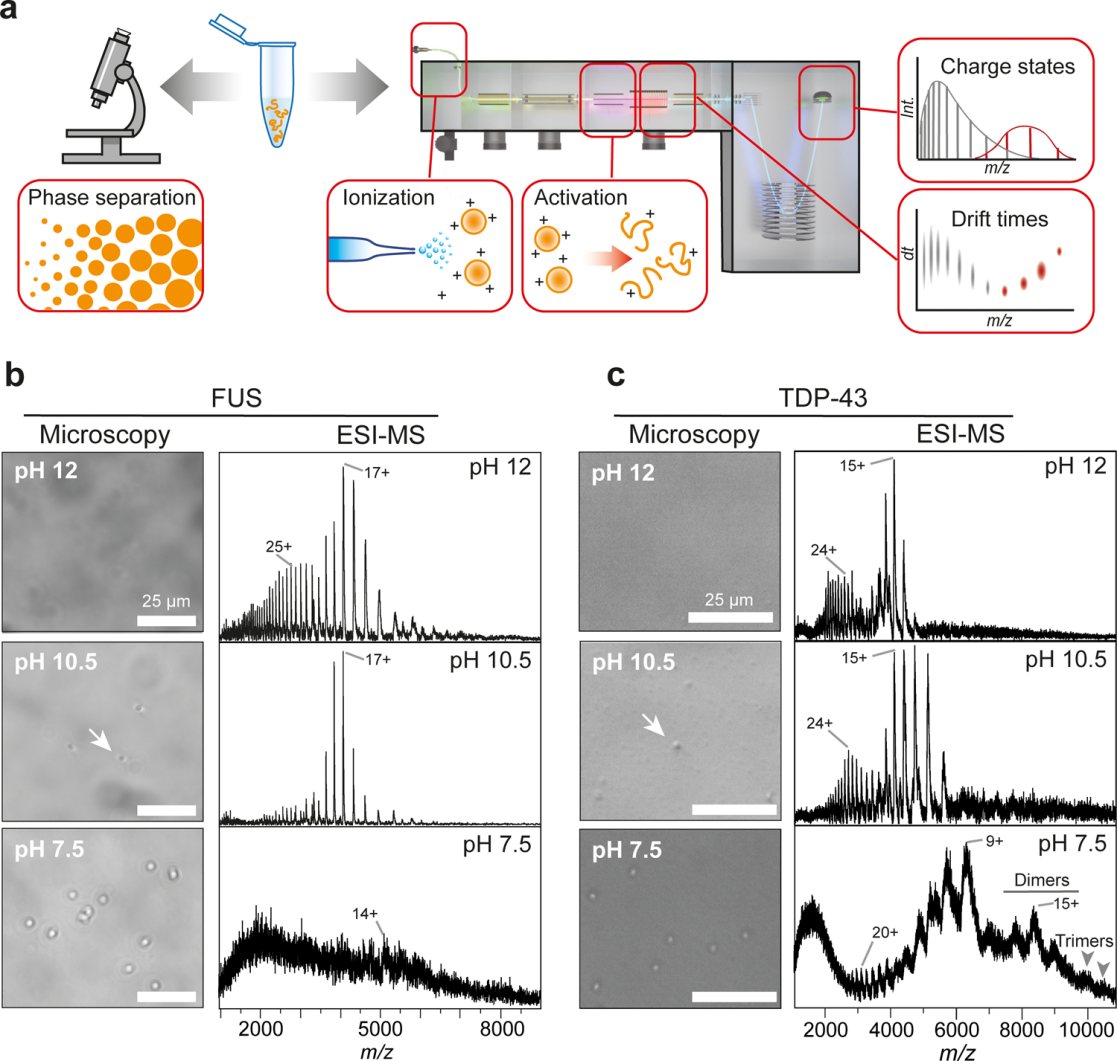
Microscopy and MS of NT*-tagged FUS and TDP-43 under denaturing and LLPS conditions. (a) Summary of the combined microscopy and nMS approach. NT*-tagged hnRNPs are exposed to different pH regimes under salt-free solution conditions and subjected to both bright-field microscopy and MS analysis. By combining nMS with IM and gas-phase dissociation, it is possible to extract ion charge states and drift times for soluble and assembled hnRNPs. (b) Bright-field microscopy of FUS shows the onset of droplet formation at pH 10.5 (arrow) and from nMS it appears as complete LLPS at pH 7.5. nMS reveals a shift from a broad, bimodal to a narrow, monomodal CSD between pH 12 to 10. At pH 7.5, low-intensity peaks corresponding to monomeric FUS can be detected by nMS. (c) TDP-43 forms a few vaguely defined droplets at pH 10.5 (arrow) and undergoes complete LLPS at pH 7.5. nMS shows a bimodal CSD at pH 12 and 10.5. At pH 7.5, a pronounced shift to lower charges occurs, and peaks corresponding in mass to dimers and trimers (arrows indicate the 19+ and 18+ ions of the TDP-43 trimer) can be detected.

We asked how LLPS of severely aggregation-prone proteins is controlled in nature. Major ampullate spidroins (MaSp), the proteins that make up spider dragline silk, contain an N-terminal domain (NT) that prevents amyloid-like aggregation but does not interfere with spidroin LLPS.^28,29^ We found that fusion of hnRNPs to a charge-engineered NT domain (NT*) prevented unwanted aggregation yet did not have a discernable effect on LLPS (see the SI for details on design and characterization of NT* fusion proteins). Having established production of full-length NT*-FUS and NT*-TDP-43 fusion proteins under native conditions, we then asked whether we could observe specific structural changes associated with LLPS. For this purpose, we turned to nMS. In nMS, intact protein complexes are ionized through electrospray ionization (ESI) and gently transferred to the gas phase for mass measurements without disturbing noncovalent interactions.^30^ The number of charges that is acquired during ESI correlates with the surface area and flexibility of the protein in solution.^31,32^ By combining nMS with IM, we can determine the collision cross section (CCS) of the ionized proteins, and in this manner obtain insights into the conformational preferences and relative stabilities of disordered proteins in the gas phase, and how they are related to solution structures.^31,33^ We have previously used nMS to reveal soluble intermediates in spider silk formation.^34^ Like silk assembly, LLPS can be induced in the absence of salt^35^ and controlled by adjusting the solution pH.^24^ As LLPS and nMS additionally require a similar protein concentration in the low micromolar range, we devised a two-pronged approach in which we prepared NT*-tagged hnRNPs at different pH regimes, monitored LLPS formation by bright-field microscopy, and subjected the same samples to nMS analysis (Figure 1a). As solvent system, we chose water/ammonia, which has been shown to be suitable for preparation of the aggregation-prone TDP-43.^36^ Importantly, the same system is well suited to study protein folding by nMS. Unlike acidic conditions, alkaline pH does not induce additional unfolding during ESI, allowing for an accurate assessment of a protein’s folded states in response to pH.^37^

To test whether we can detect proteins in liquid droplets by MS, we chose NT*-tagged FUS, since FUS is often used as a model system for LLPS. Starting at pH 12 (Figure 1b), microscopy showed no discernible structures, as expected from a visible soluble fraction after 5d at pH > 9 (Figure S1). nMS analysis revealed well-resolved peaks with a bimodal charge state distribution (CSD) centered on the 25+ and 17+ ions. Broad CSDs are indicative of conformationally flexible proteins, where extended states give rise to highly charged ions, while compact states preferentially acquire low charges.^33^

As the next step, we selected pH 10.5, close to the p*K*_a_ of tyrosine, the major driver of LLPS of FUS.^20^ At pH 10.5, we observed by microscopy very few spherical assemblies with poor contrast, possibly representing the onset of droplet formation. Strikingly, the mass spectrum showed a pronounced shift toward a monomodal CSD around the 17+ ion with a decrease in pH, while the higher-charged distribution disappeared. At pH 7.5, we detected well-resolved droplets with a diameter of around 5 μm (Figure 1b) that are morphologically indistinguishable from those formed in 20 mM Tris, 500 mM NaCl, pH 6 (Movie S1). Mass spectra show low-intensity peaks corresponding to monomeric FUS, with a charge state distribution around the 14+ ion, and significant peak broadening compared to the spectra at pH 12 and 10.5 (Figure 1b). Next, we employed collisional activation in the trap region of the T-wave ion guide, which can be used to detect polydisperse oligomers.^38^ Activation did not reveal the presence of any oligomers, resulting instead in sharper peaks and an improved signal-to-noise ratio for the monomer through adduct removal (Figure S2). We conclude that at pH 7.5, the majority of the protein is incorporated into droplets while leaving a small population of soluble monomers. These findings thus recapitulate the low saturation concentration of FUS, and the fact that around 5–10% of the protein remain in the dilute phase during LLPS.^20^

Next, we examined NT*-tagged TDP-43 (Figure 1c). As for FUS, no droplets could be observed by microscopy at pH 12. nMS showed a bimodal CSD with a compact charge state envelope around the 15+ ion, as well as highly charged ions with lower intensity, confirming the presence of flexible, monomeric protein. At pH 10.5, we observed sparse spherical assemblies by microscopy that resemble those seen for FUS. However, the bimodal CSD in nMS remained largely unchanged compared to pH 12. Upon lowering the pH to 7.5, we find TDP-43 formed well-defined droplets. Unlike FUS, however, the protein could still be detected by nMS without collisional activation. Although the CSD remained broader than for FUS, it shifted toward the higher-*m*/*z* region. We furthermore observed peaks corresponding in mass to TDP-43 dimers, as well as traces of trimers (Figures 1c and S3), suggesting the presence of oligomers in solution. Indeed, we found that around 60% of the protein remains in the dilute phase after centrifugation at pH 7.5 (Figure S3). We conclude that in contrast to the monomeric FUS, TDP-43 assembles into dimers, trimers, and possibly higher oligomers, under LLPS conditions.

### FUS and TDP-43 Exhibit Distinct Conformational Signatures during LLPS

**Figure 2:**
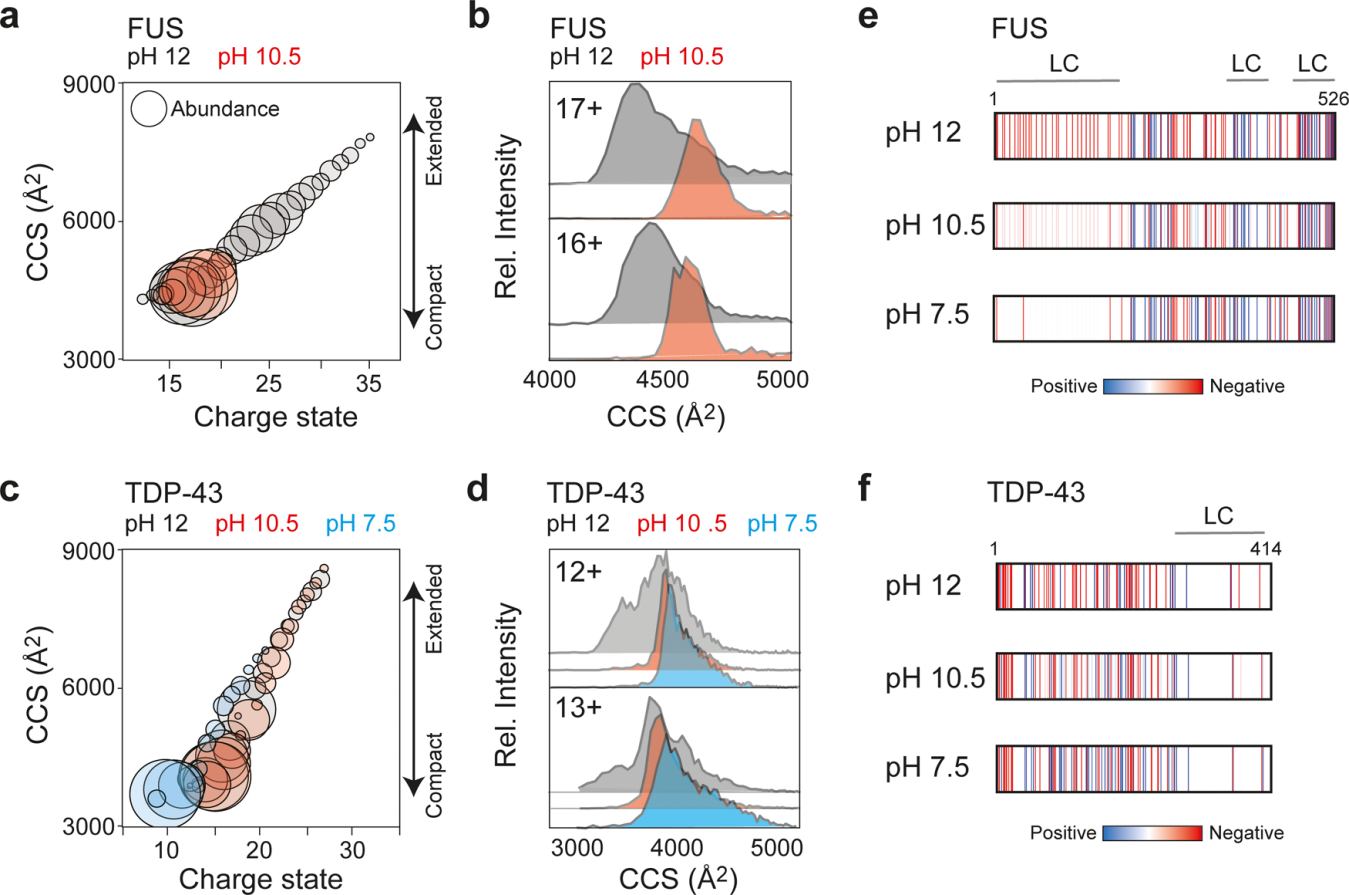
Structural changes during LLPS of NT*-tagged FUS and TDP-43. (a) Plotting the CCS of FUS as a function of charge state shows a steep rise in CCS with increasing ion charge at pH 12 (gray). At pH 10 (red), all ions display similar CCS values which are less dependent on ion charge, which is a hallmark of compact proteins. Ion abundances are indicated by the diameter of each circle. (b) CCS distributions for the 17+ and 16+ charge states of FUS are narrower at pH 10.5 compared to pH 12. The slight increase in CCS may indicate that FUS adopts a more defined structure at pH 10.5. (c) Charge state-CCS plot for TDP-43 showing a similar dependence of CCS on the ion charge for pH 12, 10.5, and 7.5, indicating no pronounced unfolded-to-globular transition. (d) CCS distributions for the 12+ and 13+ ions of TDP-43, the charge states that can be detected at all pH values, showing no pronounced changes in peak width or CCS at any condition. (e, f) Plot of the distribution of charged residues (based on their p*K*_a_) in the sequences of FUS and TDP-43 at pH 12, 10.5, and 7.5 showing the shift of the N-terminal LC domain of FUS from negative to neutral charge as the pH is lowered. TDP-43, on the other hand, does not display notable shifts in charge distributions.

We asked if the different CSDs of NT*-tagged FUS and TDP-43 are caused by conformational changes during LLPS. Previous studies have shown that the flexibility of disordered proteins in solution is reflected in the balance of Coulombic stretching and collapse that the proteins experience in the gas phase.^31^ By comparing the distributions of CCSs and charge states, we can therefore determine whether a protein is more likely to be compact (narrow CSD with near-constant CCS) or extended (broad CSD and wide CCS range) in solution. We therefore used IM-MS to determine CCSs of NT*-tagged FUS and TDP-43 at each pH value. First, we measured the CCS of FUS at pH 12. Plotting the CCS as a function of ion charge revealed a steep rise in CCS as the charge state of the protein increases (Figure 2a). This finding suggests that the CCS of the protein at alkaline pH is determined mainly by its charge, as expected for a fully disordered protein.^31^ At pH 10.5, where we observed a narrow, monomodal CSD around 17+, the CCSs remains nearly constant for all major charge states. The most abundant charge states of 16+ and 17+ display similar CCSs of 4676 and 4606 Å^2^, respectively, and considerably narrower arrival time distributions (Figure 2b). Taken together, the changes in CSD and CCS closely resemble the behavior of globular proteins at this pH,^37^ which leads us to conclude that FUS transitions from a flexible, disordered state to a compact conformation as the pH is lowered. Unfortunately, the peaks at pH 7.5 exhibited a too low signal-to-noise ratio for reliable CCS determination. The IM-MS data are thus in agreement with recent NMR and EPR studies showing that FUS undergoes significant compaction as it approaches LLPS^39^

We then used the same strategy to analyze TDP-43 at pH 12, 10.5, and 7.5. At pH 12 and 10.5, the CCS-charge state plot reveals that the protein’s cross section increases with ion charge in a similar manner as FUS at pH 12 (Figure 2c). At pH 7.5, we detected lower charge states and CCSs, but also a population with high charges and CCSs. Furthermore, the 12+ and 13+ ions, the lowest charge states present in all conditions, had similarly low CCSs, with only moderately narrower arrival time distributions at low pH (Figure 2d). From the CSD and CCS data, we conclude that TDP-43 does not undergo a pronounced unfolded-to-globular transition like FUS. Instead, its response to lowered pH is consistent with the behavior of a partially disordered protein.^37^ Interestingly, we also detected higher oligomeric states for TDP-43 at pH 7.5 that were absent in FUS. To obtain more structural information on oligomerization, we further examined the dimer by IM-MS. The CCS of the dimer was found to be 6812, 7009, and 7143 Å^2^ for the 14+, 15+, and 16+ charge states, respectively. By combining CCS values with oligomeric state and molecular weight information to mine the PDB, it is possible to extract likely complex shapes.^40^ However, this strategy did not yield a clear preference, but rather suggests a range of oblate or prolate shapes (Figure S3). Taken together, we conclude that monomers and oligomers of TDP-43 retain some conformational heterogeneity.

The IM-MS data suggest that NT*-tagged FUS and TDP-43 display distinct structural features in IM-MS as they approach the LLPS regime: FUS undergoes significant compaction around pH 10.5 and is increasingly incorporated into LLPS assemblies as the pH is lowered to 7.5. TDP-43, on the other hand, remains flexible, but shows stepwise oligomerization at pH 7.5. Barran and co-workers have reported that the conformations of disordered proteins in nMS are largely governed by charge pattern.^41^ We therefore computed the distribution of charged residues along the FUS and TDP-43 sequences at each pH (Figure 2c). We find that the LC domain of FUS, which is rich in tyrosine, undergoes a shift from negative to neutral charge as the pH drops below the p*K*_a_ of tyrosine at 10.4. For TDP-43, on the other hand, we do not observe pronounced changes in charge pattern, since both positively and negatively charged residues are distributed relatively evenly throughout the sequence.

### Structural Modeling Reveals Assembly Mechanisms

**Figure 3:**
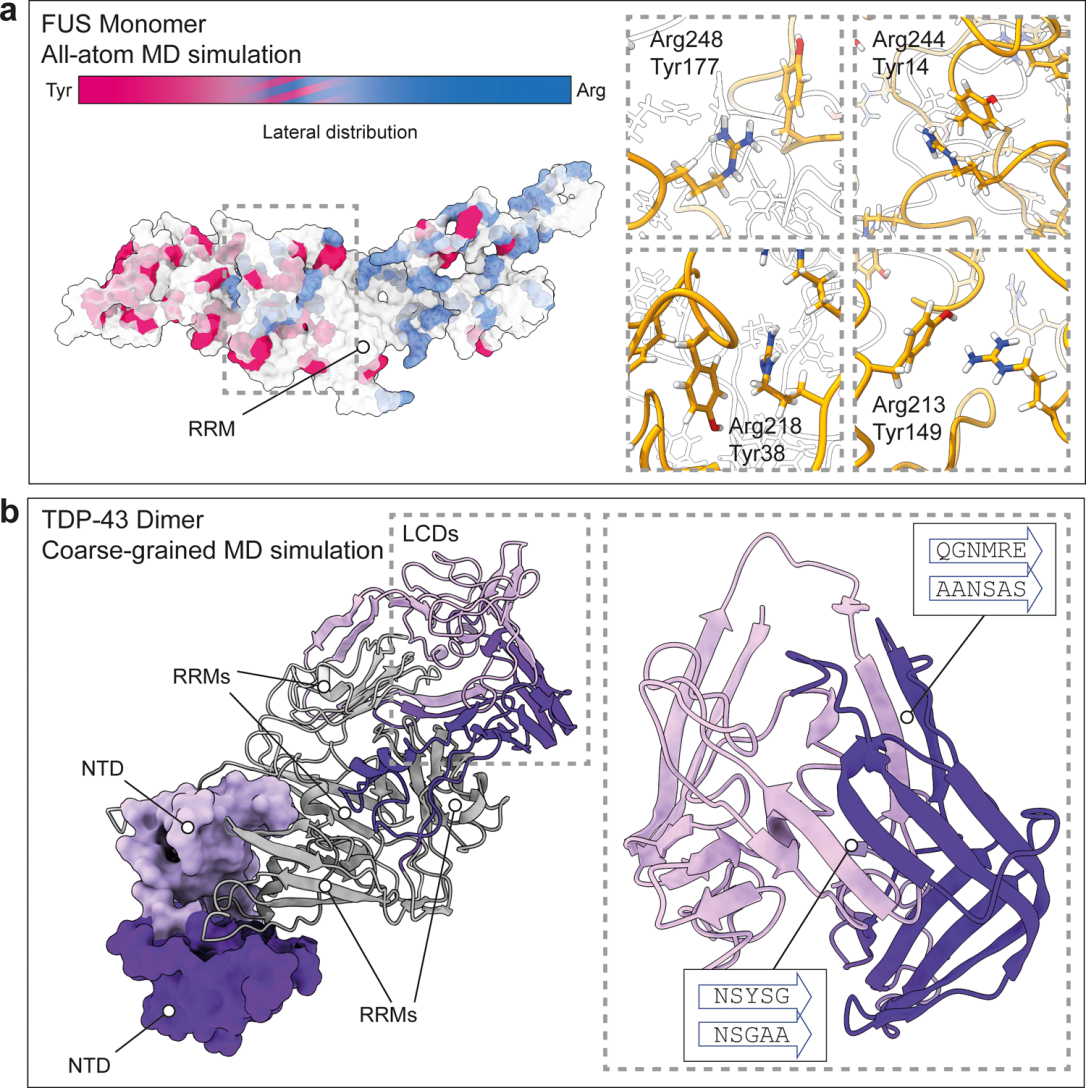
Models of FUS and TDP-43 species observed by MS. (a) All-atom MD of monomeric FUS showing a compact conformation and a bipartite organization with a tyrosine- and an arginine-rich domain. While most arginine and tyrosine residues are located at the surface, we also detect contacts between N- and C-terminal LCDs mediated by Arg-Tyr interactions, indicated by a dashed box. (b) Representative example of the AF2- and coarse-grained MD-derived model of dimeric TDP-43. The end structure from two rounds of cooling shows tight interactions between the folded NTDs (shown as surface representations). The RRMs (gray) are in proximity mediated by intermolecular contacts between the LCDs. Inset: Representative LCD interactions between two protomers reveal extensive inter- and intramolecular β-sheet formation. Sequences engaged in the intermolecular interactions are indicated in boxes.

To understand how the specific structural preferences of FUS and TDP-43 mediate self-assembly, we devised a hybrid strategy combining AlphaFold2 (AF2) structure prediction and molecular dynamics (MD) simulations (Figure S4). For FUS, we used AF2 to obtain full-length models of monomeric FUS, which we subjected to all-atom MD simulations in solution at pH 7.5. Starting from an extended, random conformation generated by AF2, FUS adopts a partially compact structure with intact RRM during a 500 ns simulation in solution (Figure 3a). Strikingly, the tyrosine-rich N-terminal LCD and the glycine/arginine-rich C-terminal LCDs remain mostly segregated in the model. The resulting structure shows distinct lateral distribution of arginines and tyrosines, with a Tyr- and an Arg-rich pole. Most tyrosines and arginines are located at the surface of the protein. However, we also found intramolecular contacts at the intersection near the RRM. We detected contacts between Tyr 14, 38, 148, and 177 with Arg 244, 218, 213, and 248, respectively. These interactions likely contribute to the compaction of the protein in solution by connecting the N- and C-terminal LCDs, with the RRM sandwiched in between.

Next, we developed models of monomeric, dimeric, and trimeric TDP-43 using coarse-grained MD simulations in solution at pH 7. Since TDP-43 is known to oligomerize via its N-terminal domains,^42,43^ we used AF2 to build NTD dimers and trimers, which we used to align the NTDs of monomeric TDP-43 models derived from SAXS measurements.^36^ To extract structural preferences, we cooled the models of full-length TDP-43 monomers, dimers, and trimers from 400 to 300 K with 20 replicates, under AWSEM force field.^44^ These first-generation models were subjected to a second round of cooling, and the resulting second-generation models were used to compute contact maps and electrostatic energies (Figure S4). Comparison of the 20 end structures reveals a significant degree of compaction but no convergence to a single conformation. The contact maps show that the RRMs interact with the NTDs and LCDs, but there are almost no contacts between NTDs and LCDs (Figure S4). The fold and interactions of the NTDs are preserved in all models (Figures 3b and S4), suggesting that the NTDs can mediate oligomerization of full-length TDP-43. Strikingly, the C-terminal LCDs consistently fold into β-sheet-rich structures that bring the RRMs in dimers and trimers into proximity (Figures 3b and S4). The C-terminal segments involved in β-sheet formation vary between replicates but always include prion-like sequences with high aggregation propensity (Figure 3b). Interestingly, in the trimer, we find that two neighboring LCDs interact, whereas the third LCD points, which is probably a result of the curvature of the NTD trimer (Figure S4). The orientation of RRMs and LCDs varies between replicates, which indicates significant flexibility, in agreement with the IM-MS results (Figure S4). Lastly, we calculated the electrostatic energies of the end structures, and found them to be slightly more favorable in the oligomers at neutral pH (Figure S4). This difference suggests that NTD interactions, which are charge-based,^43^ are favorable for TDP-43. Interactions between LCDs, on the other hand, occur almost exclusively in chargeless regions and are therefore pH insensitive. Taken together, the models reveal that TDP-43 forms flexible oligomers mediated by well-defined protein–protein interactions between NTDs and low-specificity contacts between LCDs.

### Conformational Balance of hCPEB3 Favors Aggregation over LLPS

**Figure 4:**
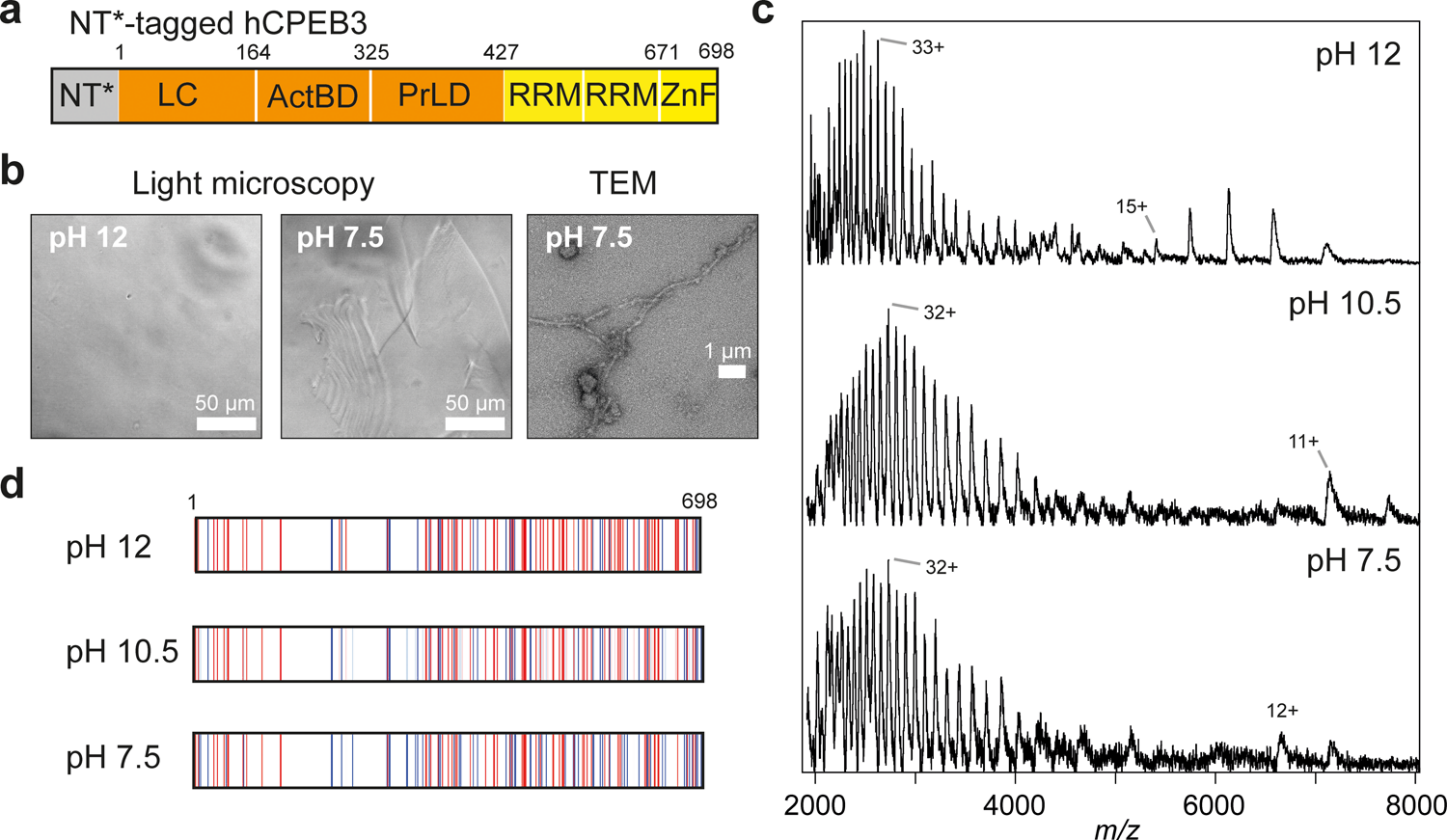
NT*-tagged hCPEB3 remains disordered and undergoes aggregation at pH 8. (a) Architecture of NT*-hCPEB3. LC, low-complexity region; RRM, RNA recognition motif; ZnF, zinc finger; ActBD, actin-binding domain; PrLD, prion-like domain. (b) Bright-field microscopy of hCPEB3 at pH 12 and 7.5 showing the appearance of elongated aggregates at low pH. TEM shows fibrillar as well as some amorphous aggregates. (c) nMS revealing highly charged monomeric hCPEB3 across the pH range tested. Note the decreased signal/noise ratio as the pH is decreased. (d) Predicted distribution of charged residues in the hCPEB3 sequence showing no pronounced local charges in response to pH.

Encouraged by these results, we turned to a less-well-understood hnRNP and asked whether nMS could reveal the structural preferences of native hCPEB3. As for FUS and TDP-43, tagging hCPEB3 (isoform 1) with NT* (Figure 4a) resulted in increased solubility, allowing us to purify the fusion protein under native conditions (Figure S5). However, NT*-tagged hCPEB3 still displayed low stability, becoming immediately insoluble if the pH was lowered below 8 (Figure 4b). To better understand how NT* affects hCPEB3 aggregation, we conducted all-atom MD simulations of NT* fused to the first 40 residues of hCPEB3 (Figure S5). We observed transient contacts between the NT* surface and the polyglutamine stretch between residues 10–26, leading us to speculate that the NT* domain may reduce, but not block, self-association of this region, and thus retard hCPEB3 aggregation. Next, we examined LLPS of NT*-tagged hCPEB3 with bright-field microscopy under the same conditions as for TDP-43 and FUS. Strikingly, we did not see spherical droplets at lower pH, but instead elongated aggregates, which stained positive for Thioflavin T (ThT), a marker for amyloid structures (Figure S5), as reported for the *Drosophila* and *Aplysia* homologues.^45,46^ In line with fluorescence microscopy, transmission electron microscopy (TEM) confirmed the presence of fibrillar aggregates (Figure 4b). nMS analysis of hCPEB3 showed a large population of highly charged ions, suggesting mostly extended protein conformations. At a lower pH, the highly charged population remained dominant, whereas the signal/noise ratio in the spectra decreased significantly (Figure 4c). The charge distribution of the N-terminal LC domain of hCPEB3, which has been identified as an aggregation hotspot, is virtually unaffected by changes in pH (Figure 4d). In fact, the observations for hCPEB3 closely mirror the behavior of amyloidogenic peptides in nMS.^47^ Our results thus indicate that despite having a similar architecture as FUS and TDP-43, hCPEB3 favors aggregation over LLPS. The fact that hCPEB3 is present in phase-separated compartments *in vivo* suggests that additional factors can control its conformational states and interactions inside cells.^17,46,48^

## Discussion

**Figure 5:**
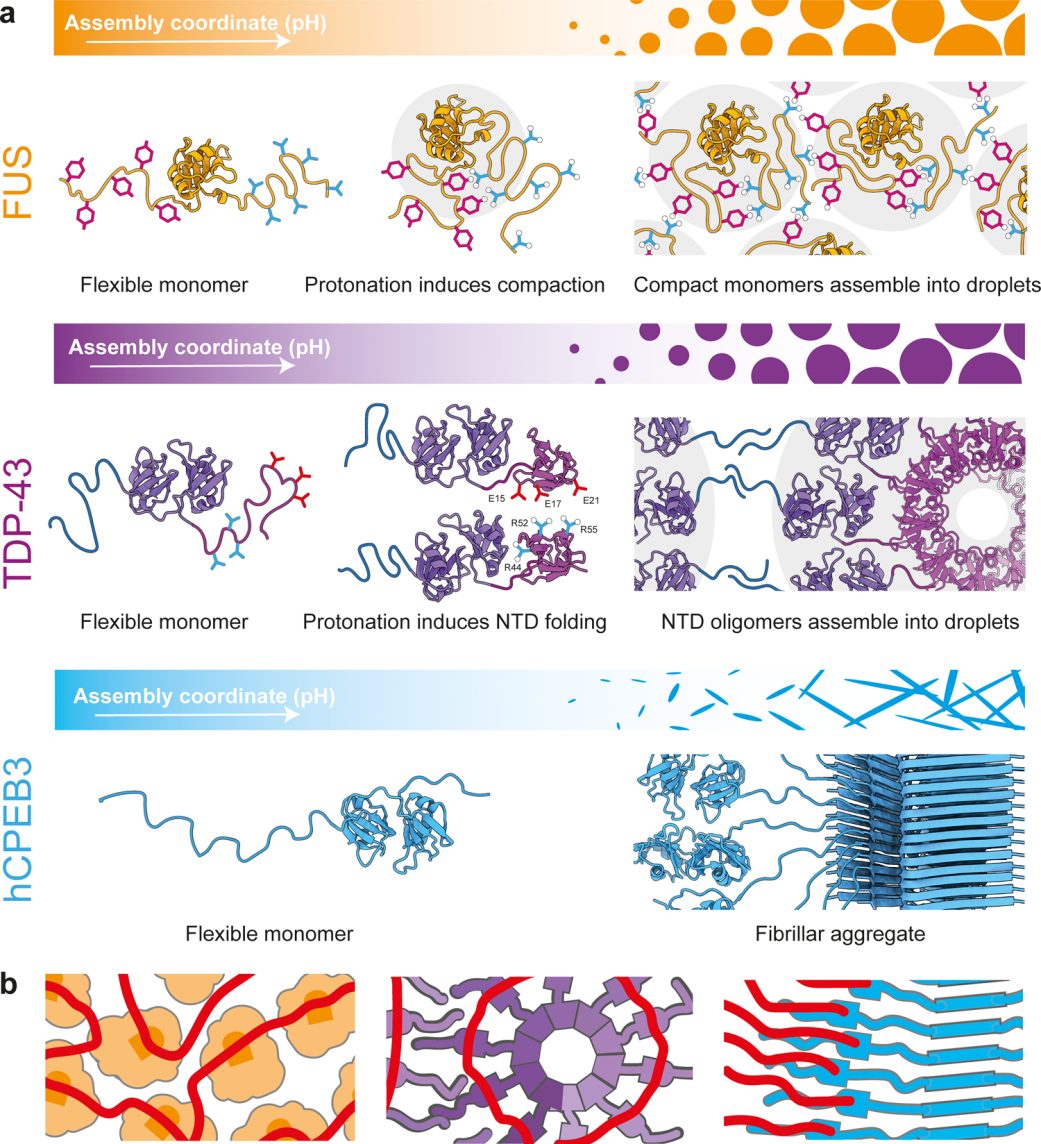
Divergent architectures and possible RNA-binding modes of FUS, TDP-43, and hCPEB3 assemblies. (a) pH-dependent assembly of FUS is accompanied by protonation of tyrosine residues and increased intramolecular interactions that lead to compaction. The compact FUS monomers can then assemble into droplets via low-specificity contacts between surface-exposed arginine and tyrosine residues. TDP-43 contains an N-terminal domain which folds at pH 10 and subsequently oligomerizes via charge interactions. Oligomerization brings the C-terminal LC domains, which do not contain titratable residues, into proximity and thus enables the interactions that drive LLPS. hCPEB3 remains mostly disordered across the pH range tested, but forms fibrillar aggregates. (b) Presumed orientation of RNAs in FUS, TDP-43, and hCPEB3 assemblies. RNA (red) binds the RRMs in compact FUS, and this aligns the protein. TDP-43 adopts a helical structure, in which neighboring RRMs bind RNA target sequences. hCPEB3 aligns its RRMs along the fibril axis that serve as anchor points for RNA molecules.

Multiple hnRNPs with similar domain organization undergo LLPS *in vitro* and *in vivo*, but insights into their assembly mechanisms remain scarce. Here, we use the NT* domain to produce the human neuronal proteins FUS, TDP-43, and hCPEB3 under nondenaturing conditions. Native IM-MS shows that all three proteins populate different conformational states when LLPS is induced by lowering the pH. Using the insights from MS to inform MD simulations, we can delineate distinct assembly mechanisms (Figure 5). Importantly, we carefully considered the potential impact of the NT* tag on LLPS and aggregation and conclude that the observed structural plasticities are specific for each hnRNP and unlikely to be affected by the NT* domain (see the SI for details). These findings demonstrate the possibility of engineering specific expression and solubility tags for LLPS applications.

FUS has a bipartite organization, with a tyrosine- and an arginine-rich domain at the N- and C-terminus, respectively. Cation-π interactions between the tyrosine and arginine residues result in low-specificity interactions that drive LLPS.^20,49^ Importantly, these interactions are affected by the protonation state of tyrosine and can be disrupted at high pH.^24,50,51^ We now find that at high pH, FUS appears disordered, but adopts a compact state as the pH approaches the physiological range. At pH 7.5, most of protein population is incorporated into droplets, but remains in equilibrium with compact monomers in the dilute phase that can be observed by nMS.^20,35^ MD simulations show that compaction of the protein results in a polar organization with an arginine- and a tyrosine-rich side. Together, these findings suggest that lowering the pH promotes cation-π interactions between the C- and N-terminal domains, which give rise to a compact, but still mostly disordered, state. The compact FUS monomers can potentially interact with other monomers via exposed tyrosine and arginine residues to form droplets (Figure 5a). Importantly, the MS and MD data are in good agreement with recent observations from NMR that FUS adopts a compact state in droplets,^39^ which underscores the validity of our approach.

In the case of TDP-43, both its folded NTD and the disordered C-terminal LCD have been implicated in LLPS.^42,43,52^ We find that phase separation of TDP-43 can be controlled by adjusting the pH, although this does not entail a clear shift in the distribution of charges along its sequence. There are virtually no titratable residues in the LCD of TDP-43, whereas the NTD requires a pH range from 10 to 5 to fold into its native structure.^53^ MS reveals the formation of dimers and trimers in a pH-dependent manner, in good agreement with the ability of the NTD to self-assemble, and supported by the presence of titratable residues at the domain interface.^42,53^ Since nMS will predominantly detect soluble complexes, we speculate that the TDP-43 oligomers are not fragments of intact droplets but rather soluble species in equilibrium with the condensed state. The importance of NTD polymerization for TDP-43 function suggests that the dimers and trimers represent biologically relevant assembly intermediates.^42,43^

MS and MD simulations suggest that the protein, unlike FUS, remains at least partially flexible upon oligomerization, in line with the LCD being extended away from the folded pats of the protein. In the MD simulations, we observe a strong tendency for β-strand formation in the LCD, which again correlates well with the high fibrillation propensity of peptide fragments from this region.^54^ TDP-43 thus appears to adopt a segmented structure in droplets, where the NTDs form a scaffold from which the LCDs protrude to engage in low-specificity contacts within the same, or between different, oligomers (Figure 5a).

hCPEB3 exhibits yet another set of characteristics: nMS suggests that the protein remains fully flexible regardless of pH but is increasingly incorporated into fibrillar aggregates as the pH approaches the physiological range. Importantly, hCPEB3 has been suggested to undergo LLPS only in the presence of crowding agents,^18^ which may indicate a strong preference for ordered aggregation over disordered interactions. Its biological function as an engram of memory formation has early on been coupled to its assembly into stable structures which sequester mRNAs and thus produce a lasting impact on the cell’s translational profile.^55,56^ The high aggregation propensity indicates that the structure of hCPEB3 may be regulated by physical modifications such as SUMOylation, rather than weak interactions between disordered regions (Figure 5a).

nMS and MD not only inform about the respective assembly mechanisms of FUS, TDP-43, and hCPEB3, but also help to delineate the locations of their RRMs (Figure 5b). For FUS, the MD simulations do not suggest a specific orientation of the RRM in the compact state, and interactions between monomers would also be unlikely to point the RRMs into a specific direction. We therefore speculate that the orientations of FUS molecules in the droplet state would be dictated by the direction of bound RNAs, rather than the other way around. However, a different picture emerges for TDP-43. The “corkscrew” structure dictated by the N-terminal domains (Figure S4) would align the RRMs of neighboring protomers, which is also evident from our MD simulations. It was recently revealed that TDP-43 assembles into multimers along its target RNA sequences.^57^ In this scenario, NTD oligomerization could stabilize the protein-RNA complex in an extended state, while contacts between the LCDs promote their condensation into droplets, and potentially affect the accessibility of the bound RNAs. hCPEB3, on the other hand, forms fibrillar structures. The highly ordered nature of these assemblies implies that the RRMs are extended out from the fibril core, which was originally proposed by Kandel and co-workers for apCPEB. hCPEB3 may thus align its target RNAs and in this manner control their translation, a process known as vectorial channeling.^58^

Here, we demonstrate that nMS can help to elucidate the molecular details of LLPS by capturing structural features of soluble species that are in equilibrium with the insoluble condensate. The insights that can be gleamed from nMS thus resemble those obtained for other insoluble protein systems such as amyloid fibrils.^59^ Our findings also highlight the fact that hnRNPs, despite being superficially similar RNA-binding proteins with disordered low-complexity domains, have evolved distinct assembly structures. By avoiding a one-size-fits-all mechanism, hnRNPs can provide scaffolds with different properties, such as the induction of different RNA conformations, in a highly specific manner.

## Experimental Section

Full experimental methods are given in the Supplementary Information File.

## Supporting Information Available

Supplementary text, design, and characterization of NT*-tagged hnRNPs; Materials and Methods; Purification and characterization of NT*-tagged FUS and TDP-43 (Figure S1); Bright-field microscopy and nMS of NT*-tagged FUS and TDP-43 assemblies (Figure S2); Characterization of NT*-tagged TDP-43 (Figure S3); Simulations of TDP-43 oligomers (Figure S4); Characterization and interactions in NT*-tagged hCPEB3 (Figure S5) (PDF)
Bright-field microscopy of droplets of NT*-FUS in 500 mM NaCl (Movie S1) (AVI)

## Supplementary Material

ja3c00932_si_001.pdf

ja3c00932_si_002.avi
