# Supplementary material for: Mass Spectrometry of RNA-Binding Proteins during Liquid–Liquid Phase Separation Reveals Distinct Assembly Mechanisms and Droplet Architectures
Source: J Am Chem Soc. 2023 May 5;145(19):10659–68. doi: 10.1021/jacs.3c00932 (PMC10197120; doi:10.1021/jacs.3c00932)
Supplement: Supplementary file 1 — ja3c00932_si_001.pdf [file ja3c00932_si_001.pdf]

Supporting Information for

Mass spectrometry of RNA-binding proteins during liquid-liquid phase separation reveals distinct assembly mechanisms and droplet architectures

Cagla Sahin*, Aikaterini Motso, Xinyu Gu, Hannes Feyrer, Dilraj Lama, Tina Arndt, Anna Rising, Genis Valentin Gese, B. Martin Hällberg, Erik G. Marklund, Nicholas P. Schafer, Katja Petzold, Kaare Teilum, Peter G. Wolynes, and Michael Landreh*

* Email: Cagla.Sahin@ki.se or Michael.Landreh@icm.uu.se or

This PDF file includes:

Supplementary Text
Figs. S1 to S5
Movie S1

Supplementary Text

Design and characterization of NT*-tagged hnRNPs

For cues on how to purify and study hnRPNs under native conditions without unwanted aggregation into fibrils, we turned to spider silk. Interestingly, the amino acid compositions of the LC regions of FUS and TDP-43 closely resemble that of MaSp1, except for poly-Ala blocks that are specific for spidroins (Figure S1a). Artificial spider silk proteins can assemble into liquid droplets *in vitro* [1], suggesting that they can suppress aggregation without interfering with LLPS. We tested this hypothesis using an artificial mini-spidroin (miniMaSp1) composed of a regulatory N-terminal (NT) domain, which cross-links the fibers at low pH, a C-terminal domain which acts as aggregation switch, and two LC repeats (Figure S1b) [2]. Brightfield microscopy in phosphate buffer reveals that at pH 7.5, miniMaSp1 forms spherical droplets with a diameter of 4-8 μm, in agreement with previous reports of LLPS by mini-spidroins [1]. The NT domain, albeit highly soluble on its own [3], does not appear to inhibit droplet formation. We therefore used a charge-reversed NT mutant (D40K/K65D), termed NT*, which does not cross-link at low pH and is a highly efficient expression tag for amyloidogenic proteins [4], to enable the production of soluble full-length hnRNPs without interfering with LLPS *in vitro.* Importantly, the PLAAC server, which predicts prion-like sequences, predicts that NT* is devoid of motifs associated with LLPS (Figure S1c). We found that recombinant expression in *E. coli* yielded high amounts of predominantly soluble NT*-tagged FUS and NT*-tagged TDP-43 (Figure S1d). We observed loss of soluble protein after incubation in common buffers, but good solubility in water, in line with previous findings (Figure S1e) [5]. We screened pH stability by incubating the proteins in H_2O at pH values ranging from 6 to 11 and monitored the formation of insoluble aggregates by centrifugation and SDS PAGE (Figure S1f). We found that the fusion proteins are stable in H_2O / ammonia or H_2O / imidazole at pH regimes above their pI. Cleavage of the NT* domain resulted in immediate loss of FUS compared to the input material (Figure S1g), confirming that NT* reduces aggregation of the purified protein.

We then asked whether the purified proteins retain natively folded RNA-binding domains. Denaturing polyacrylamide gel electrophoresis (PAGE) of natively purified FUS revealed a large amount of co-purified nucleic acids which were sensitive to RNase A treatment by

reduction of overall intensity of the band, indicating that the protein retains bound RNAs from the expression host (Figure S1h), confirming that the RNA binding capacity was at least partial intact. In the following, residual RNA was reduced by a 1 M NaCl washing step. To test the ability of the fusion proteins to undergo LLPS, we diluted NT*-FUS from a pH 9 dH_2O stock into 20 mM Tris pH 6, 500 mM NaCl, conditions under which full-length FUS undergoes phase separation [6] and monitored it with microscopy. We clearly observed spherical droplets that fuse on a minute timescale (Movie S1), which demonstrates that LLPS of NT*-tagged FUS purified under native conditions can be triggered by adjusting buffer conditions and is not inhibited by the NT*-tag.

Effects of NT* on LLPS of hnRNPs

As shown above, NT* enables the purification of hnRNPs under non-denaturing conditions. Previous work from us and others has established that the NT* domain is an efficient solubility tag [4,7,8]. Intracellular hydrogen/deuterium exchange studies have revealed that NT* remains folded even if fused to aggregation-prone proteins, thus preventing the formation of insoluble aggregates [9]. The proteins included here display different aggregation propensities *in vitro*. TDP-43, for example, aggregates upon tag removal [10], whereas hCPEB3 is soluble only under denaturing conditions [11]. Fusion to the NT* domain enables us to purify and study all three proteins under identical solution and nMS conditions, but also raises the question to what extent it affects their structures and self-assembly. In TDP-43, the NT* domain is connected to a folded NTD via a short, disordered segment composed of the TEV cleavage site and the first 4-5 residues of TDP-43. We detect by nMS dimerization and trimerization of NT*-tagged TDP-43 at pH 7.5, as predicted for isolated TDP-43 NTDs. NT*, on the other hand, does not oligomerize [4]. We thus conclude that our observations can be attributed to specific oligomerization of TDP-43, although we cannot exclude that the NT* domain may influence the efficiency of the process. Recently, we found that NT* induces compaction of the first 60 residues of the disordered transactivation domain of p53 by binding to short hydrophobic segments [12]. As for p53, NT* is in hCPEB3 and FUS connected to a disordered N-terminal domain; however, the disordered NTD of FUS is three times longer than that of p53 (270 *vs.* 90 residues) and has a significantly lower hydrophobicity. We thus consider it highly unlikely

that the near-complete unfolded-to-globular transition we observe in FUS can be attributed to the presence of the NT* domain. For hCPEB3, we do not observe any compaction comparable to that of p53 or FUS, which agrees with the very transient contacts observed in MD simulations (Figure S5). Based on this evidence, we conclude that the NT* domain reduces the aggregation propensity of hnRNPs through steric hindrance and its own high solubility but does not prevent low-complexity domains from forming transient interactions that mediate LLPS. As a result, the different structural plasticities observed here are specific for each protein.

Materials and Methods

Reagents

All chemicals were purchased from Sigma unless noted otherwise.

Protein production

MiniMaSp1 was produced as described [2]. Constructs containing a N-terminal NT* tag with 6-His repeat and a TEV cleavage site followed by either FUS, TDP-43 or hCPEB3 were ordered from Genscript. The plasmids were transformed into BL21(DE3) cells (Sigma) using heat-shock. Cells were grown in LB medium at 37°C and induced with IPTG at a OD_{600} at 0.6-0.8. After induction protein was expressed overnight at 18°C for NT*-FUS and NT*-TDP-43, and at 12°C for NT*-hCPEB3. Cells were harvested by centrifugation at 4000 rpm for 20 min at 4°C and stored at -80°C until purification. Prior to purification the cell pellet was thawed on ice and resuspended in 10 mL lysis buffer (dH_2O with 10 mM imidazole, pH 9, 0.1 mg/mL lysozyme and cOmplete mini EDTA-free protease inhibitor tablets (Roche)) per 0.25 L culture before sonication at 50 % amplitude in 5 sec pulses for 10 minutes. Lysate was spun down at 10,000 g for 30 min at 4 °C, and the supernatant was loaded on a HisTrap column equilibrated in water with 10 mM imidazole, pH 9, washed with 1 M NaCl to reduce bound nucleic acids, and eluted the protein with imidazole (250 mM). Eluted fractions were analyzed on SDS-PAGE (miniProtean TGX, BioRad) under non-reducing conditions and without boiling. Fractions containing the protein of interest were dialyzed against dH_2O prior to experiments, according to the ideal conditions identified for TDP-43 [5].

Microscopy

Protein samples in dH_2O were found to have a pH of 7.5, as measured by a micro-electrode, which is due to residual histidine from the purification. By addition of 0.12 % or 12.5 % ammonia, the pH reached 10.5 and 12, as we previously used to study proteins under alkaline conditions [13]. 50 μL of the solution were transferred to a non-binding surface 96 well black plate with clear bottom (Corning). Samples were imaged at room temperature (ca. 23°C) on a CellObserver microscope (Zeiss) with the transmitted light channel. 20x or 40x magnification was used for imaging. For imaging of amyloids with the fluorophore Thioflavin T, we simply added Thioflavin T to the sample, and added a FITC excitation and emission filter set for imaging. Zen Blue (Zeiss) and ImageJ (https://imagej.nih.gov/) was used for analysis of the images.

pH-dependent solubility assay

Concentrated NT*-tagged hnRNPs were diluted in Tris buffers with pH 6, 7, 8, 9, 10 and 11 to reach a final buffer concentration of 20 mM tris and a final protein concentration of 1 mg/mL. Samples were incubated at room temperature, where aliquots were taken out at different time points, spun down at 13000 ×g for 10 min. Supernatant and pellet samples were mixed with 4x Laemmli sample buffer (non-reducing), and samples ran on a 4-15 % stain-free TGX gel (BioRad). The gels were imaged with ChemiDoc (BioRad). The fraction of soluble TDP-43 in the MS samples was assessed by preparing 100 μL of 15 μM TDP-43 at pH 11 or 7.5. The insoluble fraction was isolated by centrifugation in a benchtop centrifuge with 12.000 × g for 10 min. The protein concentration was determined by measuring the 280 nm absorbance of the supernatant with NanoDrop (Thermo Fisher) and the soluble protein fraction at pH 7.5 calculated as fraction of the concentration at pH 12.

RNA binding assays

To assess whether NT*-FUS is co-purified with RNA, the final NT*-FUS samples (with or without a 1M NaCl wash during purification) were subjected to degradation of broadband RNase. 50 μL HisTrap elution fractions of NT*-FUS (1 μM protein, 255 mM imidazole) were first degraded using proteinase K (1 μg/μL final concentration) at 50°C for 45

minutes. After heat-denaturation of proteinase K (95°C, 15 minutes), 1 µL RNase A (30 units) was added to the NT*-FUS samples and incubated at 37°C for 30 minutes. Yeast tRNA extract (Sigma, 10 µM in 50 mM imidazole) served as a control sample. The reactions were analyzed on denaturing polyacrylamide gel electrophoresis (Mini-PROTEAN TBA-Urea Precast Gels, BioRad), run for 30 minutes at 250 V. 5 µL sample was diluted in 5 µL formamide containing bromphenol blue, heated to 95°C for 2 minutes and loaded onto the gel. The gel was stained with SYBRGold (Sigma) according to manufacturer instructions and imaged with ImageQuant 800 (Amersham).

Native and ion mobility mass spectrometry

NT*-tagged hnRNPs in dH_2O containing 0, 0.125 or 12.5 % ammonia to reach pH 7.5, 10.5 or 12.0 with a final protein concentration of 10 to 15 µM were mixed immediately prior to transfer to a nESI capillary (Thermo). Mass spectra were acquired in positive ionization mode on a Waters Synapt G1 TWIMS MS modified for analysis of intact protein complexes (MS Vision, The Netherlands), equipped with an off-line nanospray source. The capillary voltage was set to 1.5 kV. The trap voltage was set to 10 V and stepwise increased up to 100 V for collisional activation. The source temperature was 30 °C. The cone voltage was 50 V, and the source pressure was set to 8 mbar. The ion trap voltage was set to 10 V and increased in steps of 10 V to 150 V for collision induced unfolding. Trap gas was argon with a flow rate of 4 mL/h, and IMS gas was nitrogen with a flow rate of 30 mL/h. IMS settings were wave height 10 V, wave velocity 200 V for NT*-TDP-43, and wave height 11 V, wave velocity 450 V for NT*-FUS. β-lactoglobulin, BSA, and Concanavalin A (Sigma) were used as T-Wave calibrants for NT*-TDP43, and BSA only (due to the virtually identical MW) for NT*-FUS as described [14]. Data were analyzed using MassLynx 4.0 and Pulsar software packages [15].

CCS database searches

To find candidate shapes for the TDP-43 dimer, PDB searches were performed as described [16,17], using a CCS of 6990 $Å^2$ and a MW of 122 kDa as search criteria against CCS values calculated by IMPACT[18] for the entire PDB. The ten best-scoring

homodimers were extracted from the list of the 50 best matches, and the aspect ratios were calculated in UCSF Chimera [17,19].

Modelling and simulation of NT*-hCPEB3$^{1-40}$

The solution state structure of the N-terminal domain (NT) from the spider silk protein was retrieved from the Protein Data Bank (PDB ID: 2LPJ). The lowest energy state structure (Model 1) from the ensemble of NT structures was selected and two *in-silico* mutations "D40K and K65D" were incorporated (NT*) into the structure. A 47 amino acid sequence comprising of TEV cleavage site (^{1}ENLYFQS^{7}) and the first 40 residues from human CPEB3 protein (Uniprot ID: Q8NE35) was considered for modelling. Extended state conformation of TEV+CPEB3$^{1-40}$ polypeptide segment was generated using the LEaP module of AMBER18[20], and attached via a peptide bond with NT*. The NT*-TEV-CPEB3$^{1-40}$ construct was then subjected to explicit solvent MD simulations. The N- and C-terminus of the chimeric model were capped with ACE (acetyl) and NME (N-methyl) functional groups respectively. The model was placed in the centre of a truncated octahedral box whose dimensions was fixed by maintaining a minimum distance of 8 Å between any protein atom and the box boundaries. TIP3P water model [21] was used to solvate the structure. The net charge of the systems was neutralized by adding 8 Na^+ counter ions. All-atom molecular dynamics simulations were carried out using the PMEMD module through the AMBER18 suite of programs employing ff14SB force field parameters [22]. The system was energy minimized using steepest descent followed by conjugate gradient schemes, heated to 300 K over 30 ps under NVT ensemble and equilibrated for 200 ps in NPT ensemble. The final production dynamics was run for 1 µs under NPT conditions. Simulation temperature was maintained at 300 K using Langevin dynamics [23] (collision frequency: 1.0 ps^{-1}) and the pressure was kept at 1 atm using weak-coupling [24] (relaxation time: 1ps). Periodic boundary conditions were applied on the box edges and Particle Mesh Ewald (PME) method [25] was used to compute long range electrostatic interactions. SHAKE algorithm [26] was used to constrain all the bonds involving hydrogen atoms and the equation of motion was solved with an integration time step of 2 fs.

Modelling and simulation of FUS

The initial full length unrelaxed structure of FUS (526 residues; Uniprot ID: P35637) was generated using Alphafold2 (AF2) through the jupyter notebook for Colabfold [27] using the default parameters and no template. The AF2 generated protein model was then placed in the centre of a truncated octahedral box whose dimensions was fixed by maintaining a minimum distance of 6 Å between any protein atom and the box boundaries. OPC [28], which is a 4-point rigid water model was used to solvate the structure and the net charge of the systems was neutralized by adding 14 Cl^- counter ions. The final system consisted of a total of 251 160 atoms. All-atom molecular dynamics simulations were carried out using the PMEMD module through the AMBER18 suite of programs employing ff19SB force field parameters [29]. The system was energy minimized using steepest descent followed by conjugate gradient schemes, heated to 300 K over 30 ps under NVT ensemble and equilibrated for 200 ps in NPT ensemble. The final production dynamics was run for 500 ns under NPT conditions.

Modelling and simulation of TDP-43

The initial full length structure of TDP-43 monomer was the SAXS-derived MD model (418 residues), provided by Prof. Samar Hasnain, Liverpool. AF2 was used to generate oligomeric structures of the TDP-43 N-terminal domains (NTD). Then the full-length monomer model was fitted into the AF2 structures of NTD oligomers to generate full length TDP-43 dimers and trimers. Coarse-grained molecular dynamics simulations for TDP-43 monomer, dimer or trimer were carried out using the AWSEM force field [30], running in openMM with Langevin integrator with a time step of 2 fs. AWSEM annealing was iterated twice to achieve a consistent prediction. In each annealing generation, simulations started with the same initial structures and were run 20 times with different random seeds. For the 1st generation, systems cooled down from 400K to 300K in $6x10^6$ steps (around 12 μs in laboratory time). Then, the last frames of annealing were relaxed for another $6x10^6$ steps at 300K. For the 2nd generation, those contacts that were formed more than 6 times in the 20 structures from the last frames of the relaxing simulations in the 1st generation, were then used as constraints during annealing. Here, a contact refers to a residue pair with their CA atoms distance smaller than 9.5 Å. The annealing and

relaxing protocol was the same as for the 1st generation runs. Finally, the last frames of the 2nd generation runs became the end structures. Domain contact maps (Fig. S4b) were calculated by counting the average number of contacted residue pairs between TDP-43 domains over 20 end structures. The average and standard variance of electrostatic energies per protomer for 20 end structures (Fig. S4d) were calculated using the definition of the Debye-Huckel potential in the AWSEM Hamiltonian, under different pH conditions.

Negative stain electron microscopy

For negative stain EM, 4 mg of lyophilized hCPEB3 were dissolved in ddH_2O, sonicated for 30 sec and incubated for 3 days at 37 °C. The CPEB3 samples were either diluted in water or in 10 mM HEPES pH 7.15, 75 mM NaCl, 10 mM KCl, 2 mM $MgCl_2$ and 5 mM TCEP [31] to a final concentration of 2 to 4 µM CPEB3. 5 µL was loaded to 200 mesh copper grids with Formvar/Carbon support film that had been glow-discharged at 25 mA for 30 sec. After one minute of incubation, the liquid was removed and the negative staining was carried out by applying a 5 µl of 1 % (w/v) uranyl acetate (in H_2O) to the grid for 20-30 seconds. Then, the liquid was removed and the procedure was repeated 6 times. The grids were imaged in Talos 120 C G2 (Thermo Scientific) equipped with a CETA-D detector.

References

1. Malay, A. *et al.* Spider silk self-assembly via modular liquid-liquid phase separation and nanofibrillation. *Sci. Adv.* **6**, eabb6030 (2020).
2. Andersson, M. *et al.* Biomimetic spinning of artificial spider silk from a chimeric minispidroin. *Nat. Chem. Biol.* **13**, 262–264 (2017).
3. Askarieh, G. *et al.* Self-assembly of spider silk proteins is controlled by a pH-sensitive relay. *Nature* **465**, 236–238 (2010).
4. Kronqvist, N. *et al.* Efficient protein production inspired by how spiders make silk. *Nat. Commun.* **8**, 15504 (2017).
5. Wright, G. S. A. *et al.* Purification and Structural Characterization of Aggregation-Prone Human TDP-43 Involved in Neurodegenerative Diseases. *iScience* **23**,

101159 (2020).
6. Krainer, G. *et al.* Reentrant liquid condensate phase of proteins is stabilized by hydrophobic and non-ionic interactions. *Nat. Commun.* **12**, 1085 (2021).
7. Abelein, A. *et al.* High-yield Production of Amyloid-β Peptide Enabled by a Customized Spider Silk Domain. *Sci. Rep.* **10**, 235 (2020).
8. Sarr, M. *et al.* A spidroin-derived solubility tag enables controlled aggregation of a designed amyloid protein. *FEBS J.* **285**, 1873–1885 (2018).
9. Kaldmäe, M. *et al.* High intracellular stability of the spidroin N-terminal domain in spite of abundant amyloidogenic segments revealed by in-cell hydrogen/deuterium exchange mass spectrometry. *FEBS J.* **287**, 2823–2833 (2020).
10. McGurk, L., Gomes, E., Guo, L., Shorter, J. & Bonini, N. M. Poly(ADP-ribose) Engages the TDP-43 Nuclear-Localization Sequence to Regulate Granulo-Filamentous Aggregation. *Biochemistry* **57**, 6923–6926 (2018).
11. de Mingo, D. R., López-García, P., Hervás, R., Laurents, D. V & Carrión-Vázquez, M. Molecular Determinants of Liquid Demixing and Amyloidogenesis in Human CPEB3. *BioRxiv* https://doi.org/10.1101/2020.06.02.129783 (2020).
12. Kaldmäe, M. *et al.* A ’Spindle and Thread’-Mechanism Unblocks p53 Translation by Modulating N-Terminal Disorder. *Structure* DOI:https://doi.org/10.1016/j.str.2022.02.013 (2022) doi:10.2139/ssrn.3891056.
13. Sahin, C. *et al.* Ion mobility-mass spectrometry shows stepwise protein unfolding under alkaline conditions. *Chem. Commun.* **57**, 1450–1453 (2021).
14. Bush, M. F. *et al.* Collision cross sections of proteins and their complexes: A calibration framework and database for gas-phase structural biology. *Anal. Chem.* **82**, 9557–9565 (2010).
15. Allison, T. M. *et al.* Quantifying the stabilizing effects of protein–ligand interactions in the gas phase. *Nat. Commun.* **6**, 8551 (2015).
16. Kaldmae, M., Sahin, C., Saluri, M., Marklund, E. G. & Landreh, M. A strategy for the identification of protein architectures directly from ion mobility mass spectrometry data reveals stabilizing subunit interactions in light harvesting complexes. *Protein Sci.* **28**, 1024–1030 (2019).

17. Landreh, M. *et al.* Predicting the Shapes of Protein Complexes through Collision Cross Section Measurements and Database Searches. *Anal. Chem.* **92**, 12297–12303 (2020).
18. Marklund, E. G., Degiacomi, M. T., Robinson, C. V., Baldwin, A. J. & Benesch, J. L. P. Collision cross sections for structural proteomics. *Structure* **23**, 791–799 (2015).
19. Pettersen, E. F. *et al.* UCSF Chimera—A Visualization System for Exploratory Research and Analysis. *J Comput Chem* **25**, 1605–1612 (2004).
20. Case, D. A. *et al.* Amber 18. *Univ. California, San Fr. 2018* (2018).
21. Jorgensen, W. L., Chandrasekhar, J., Madura, J. D., Impey, R. W. & Klein, M. L. Comparison of simple potential functions for simulating liquid water. *J. Chem. Phys.* **79**, 926–935 (1983).
22. Maier, J. A. *et al.* ff14SB: Improving the Accuracy of Protein Side Chain and Backbone Parameters from ff99SB. *J. Chem. Theory Comput.* **11**, 3696–3713 (2015).
23. Loncharich, R. J., Brooks, B. R. & Pastor, R. W. Langevin dynamics of peptides: The frictional dependence of isomerization rates of N-acetylalanyl-N′-methylamide. *Biopolymers* **32**, 523–535 (1992).
24. Berendsen, H. J. C., Postma, J. P. M., van Gunsteren, W. F., DiNola, a & Haak, J. R. Molecular dynamics with coupling to an external bath. *J. Chem. Phys.* **81**, 3684–3690 (1984).
25. Darden, T., York, D. & Pedersen, L. Particle mesh Ewald: An N·log(N) method for Ewald sums in large systems. *J. Chem. Phys.* **98**, 10089–10092 (1993).
26. Ryckaert, J. P., Ciccotti, G. & Berendsen, H. J. C. Numerical integration of the cartesian equations of motion of a system with constraints: molecular dynamics of n-alkanes. *J. Comput. Phys.* **23**, 327–341 (1977).
27. Mirdita, M. *et al.* ColabFold - Making protein folding accessible to all. *BioRxiv Prepr.* https://doi.org/10.1101/2021.08.15.456425 (2021).
28. Izadi, S., Anandakrishnan, R. & Onufriev, A. V. Building water models: A different approach. *J. Phys. Chem. Lett.* **5**, 3863–3871 (2014).
29. Tian, C. *et al.* Ff19SB: Amino-Acid-Specific Protein Backbone Parameters

Trained against Quantum Mechanics Energy Surfaces in Solution. *J. Chem. Theory Comput.* **16**, 528–552 (2020).

30. Lu, W. *et al.* OpenAWSEM with Open3SPN2: A fast, flexible, and accessible framework for large-scale coarse-grained biomolecular simulations. *PLoS Comput. Biol.* **17**, e1008308 (2021).

31. Hervas, R. *et al.* Cryo-EM structure of a neuronal functional amyloid implicated in memory persistence in Drosophila. *Science (80-.).* **367**, 6483 (2020).

32. Jaudzems, K. *et al.* PH-dependent dimerization of spider silk N-terminal domain requires relocation of a wedged tryptophan side chain. *J. Mol. Biol.* **422**, 477–487 (2012).

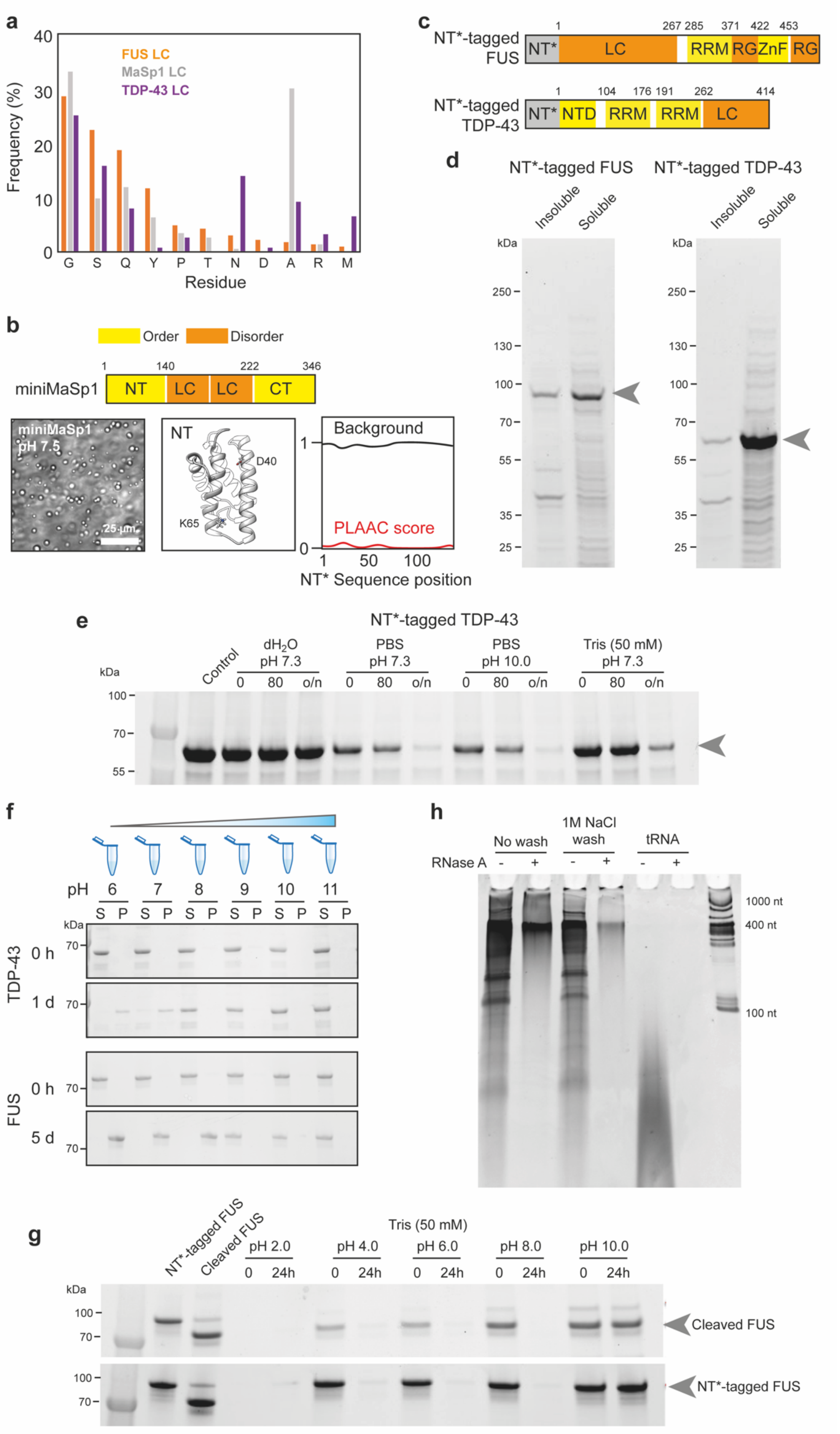
a
FUS LC
MaSp1 LC
TDP-43 LC
Frequency (%)
Residue
b
Order
Disorder
miniMaSp1
NT
LC
LC
CT
miniMaSp1 pH 7.5
25 µm
D40
K65
Background
PLAAC score
NT* Sequence position
c
NT*-tagged FUS
NT*
LC
RRM
RG
ZnF
RG
NT*-tagged TDP-43
NT*
NTD
RRM
RRM
LC
d
NT*-tagged FUS
NT*-tagged TDP-43
Insoluble
Soluble
kDa
e
NT*-tagged TDP-43
Control
dH2O pH 7.3
PBS pH 7.3
PBS pH 10.0
Tris (50 mM) pH 7.3
0 80 o/n
f
pH 6 7 8 9 10 11
S P
TDP-43
0 h
1 d
FUS
0 h
5 d
h
No wash
1M NaCl wash
tRNA
RNase A - +
1000 nt
400 nt
100 nt
g
NT*-tagged FUS
Cleaved FUS
Tris (50 mM)
pH 2.0
pH 4.0
pH 6.0
pH 8.0
pH 10.0
0 24h
Cleaved FUS
NT*-tagged FUS

Figure S1. Purification and characterization of NT*-tagged FUS and TDP-43. (a) The LC domains from FUS, TDP-43, and MaSp1 have similar amino acid compositions, except for poly-A blocks that are specific for MaSp proteins. (b) The architecture of the miniMaSp1 spidroin with NTD, N-terminal domain; LC, low complexity region, and CTD, C-terminal domain. Brightfield microscopy shows that miniMaSp1 forms spherical droplets at pH 7.5. The structure of the NT domain (PDB ID 4FBS)[32] is rendered as cartoon with the residues K65 and D40, which are swapped in the NT* variant, shown as sticks. The absence of prion-like sequences capable of LLPS (PLAAC score, red) in the NT* domain was assessed using the PLAAC server (http://plaac.wi.mit.edu). (c) Architecture of the NT*-tagged full-length FUS and TDP-43 variants used in this study. RRM, RNA recognition motif; ZnF, zinc finger. (d) NT*-FUS and NT*-TDP-43 fusion proteins expressed in *E. coli* BL21 under standard conditions (see Methods) is located predominantly in the soluble fraction following lysis by gentle sonication in dH_2O. (e) Stability of NT*-tagged TDP-43 in standard buffer shows moderate aggregation after prolonged incubation in PBS and Tris, and good stability in dH_2O. (f) NT*-tagged FUS and NT*-tagged TDP-43 resist aggregation and display long-term stability under mildly alkaline conditions. Solubility was determined by incubating protein at pH values between 6 and 11 and separating soluble (S) and insoluble (P) fractions by centrifugation. (g) Proteolytic cleavage of the NT* domain from FUS reduces its solubility. NT*-tagged FUS was either incubated directly or cleaved using TEV protease, and the soluble cleaved or uncleaved protein in tris buffer at pH 4 to 10 was isolated by centrifugation and visualized by SDS-PAGE analysis. (h) Denaturing PAGE of NT*-tagged FUS purified by affinity chromatography under native conditions reveals co-purified endogenous RNAs.

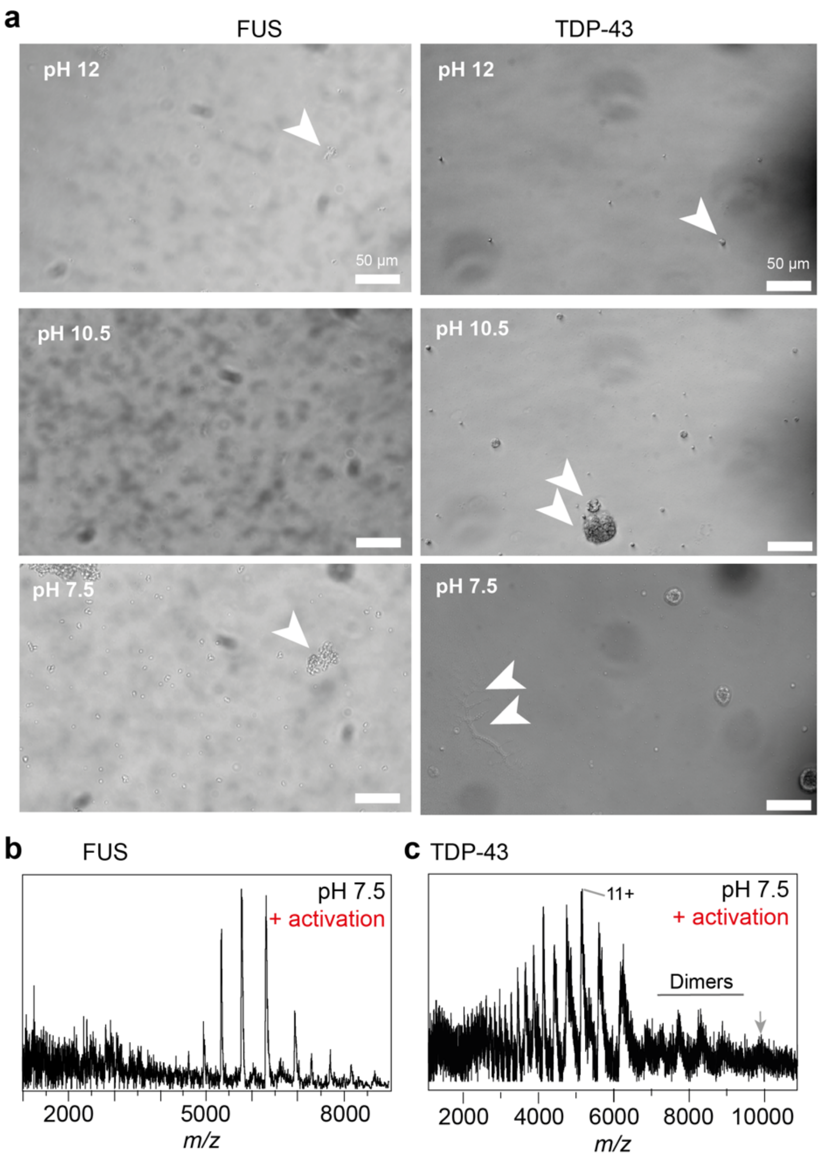

Figure S2. Bright-field microscopy and nMS of NT*-tagged FUS and TDP-43 assemblies. (a) Brightfield microscopy images of NT*-FUS and NT*-TDP-43 at pH 12, 10.5, and 7.5. We observe small amounts of amorphous aggregates (arrows) at all pH values, which likely represent protein carried over from the insoluble fraction during purification. (b) and (c) nMS spectra of FUS and TDP-43, respectively, at pH 8 with an ion trap energy of 100 V. Collisional activation increases the signal-to-noise ratio of the peaks corresponding to monomeric FUS, but do not reveal any oligomers (compare Figure 2b). Collisional activation of TDP-43 at pH 7.5 does not have a notable impact on protein charge states compared to pH 10.5 (compare Figure 2c). The arrow indicates the putative 19+ charge state of the trimer.

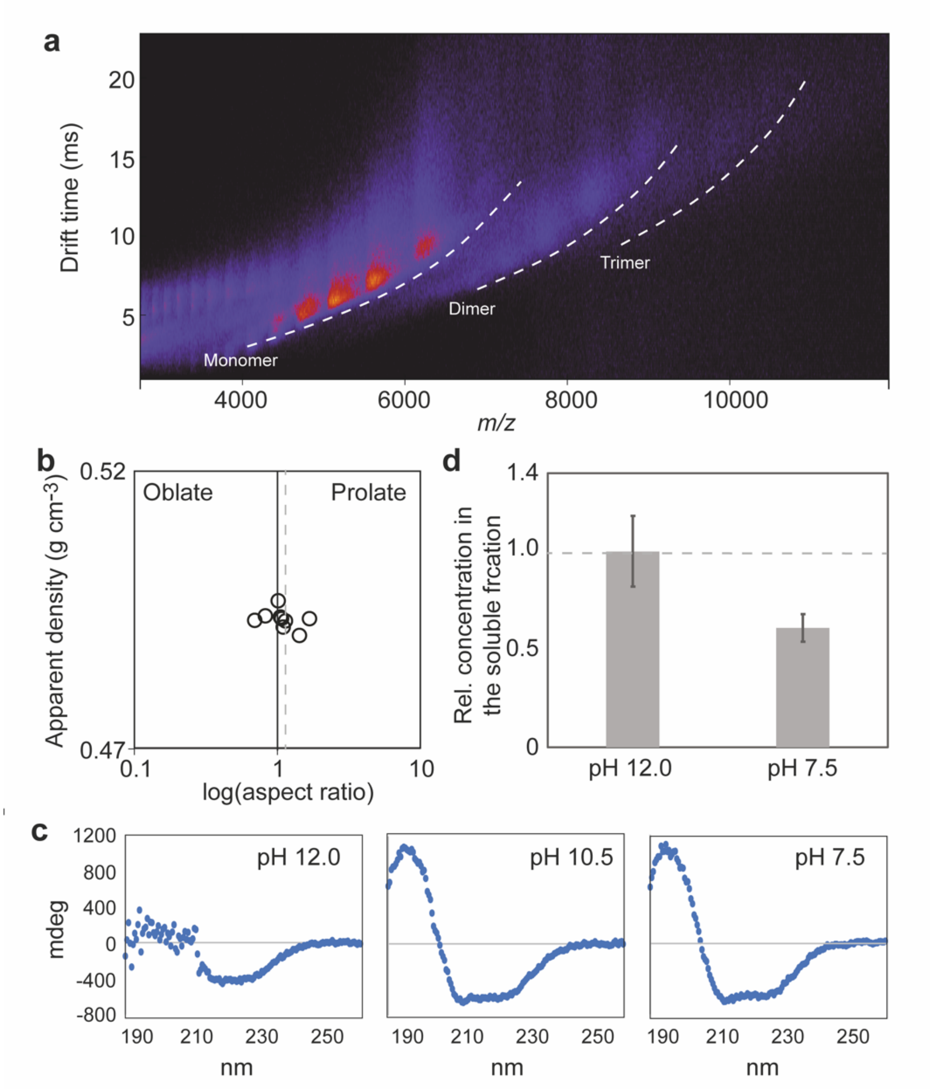

Figure S3. Characterization of NT*-tagged TDP-43. (a) The mobiligram of TDP-43 at pH 7.5 shows ion series corresponding in mass to compact monomers, dimers, and trimers. (b) Searching the PDB for protein dimers with similar MW and CCS as dimeric TDP-43 yields both oblate and prolate structures with an aspect ratio close to 1, suggesting a mixture of possible shapes for the dimer. The average aspect ratio for the top ten matches is indicated by a dashed line. (c) CD measurements of TDP-43 at alkaline and physiological pH show folding at pH 10 and below but no pronounced changes between pH 10 (onset of droplet formation) and pH 7.5 (complete droplet formation), which indicates that LLPS occurs after the protein has folded. (d) The fraction of soluble TDP-43 at pH 12 and pH 7.5 was determined by centrifugation. Error bars indicate the standard deviation of three independent repeats.

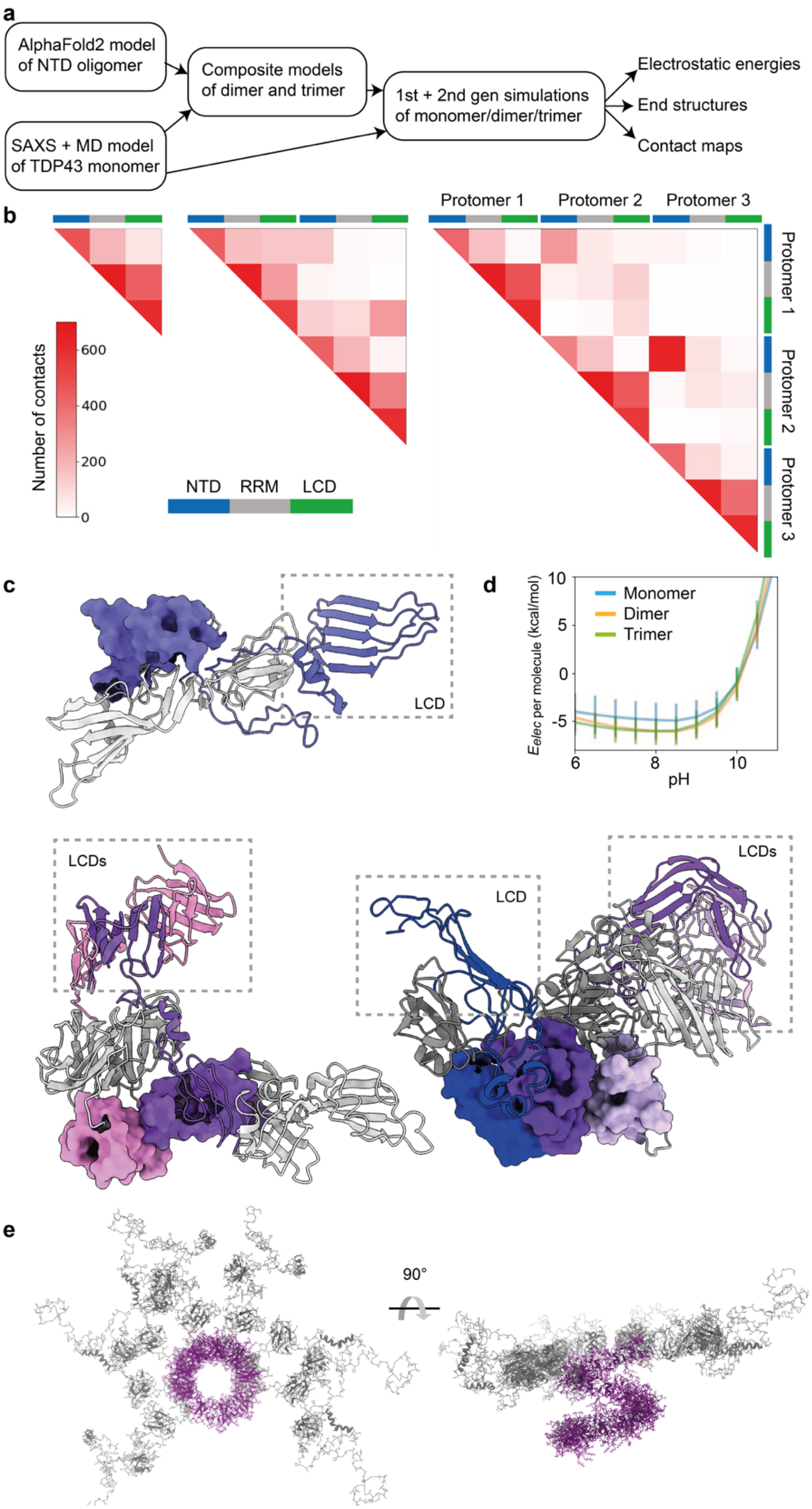
a
AlphaFold2 model of NTD oligomer
SAXS + MD model of TDP43 monomer
Composite models of dimer and trimer
1st + 2nd gen simulations of monomer/dimer/trimer
Electrostatic energies
End structures
Contact maps
b
Protomer 1
Protomer 2
Protomer 3
Number of contacts
600
400
200
0
NTD
RRM
LCD
c
LCD
LCDs
LCD
LCDs
d
Monomer
Dimer
Trimer
E_{elec} per molecule (kcal/mol)
10
5
0
-5
6
8
10
pH
e
90°

Figure S4. Simulations of TDP-43 oligomers. (a) The computational strategy employed to generate TDP-43 monomer, dimer, and trimer structures. Briefly, AF2 was used to generate oligomeric structures of the TDP-43 NTD. SAXS-derived MD models (kindly provided by Prof. Samar Hasnain, Liverpool) were then fitted into the NTD oligomers to generate dimers and trimers. Monomers, dimers, and trimers were subjected to two rounds of cooling and relaxing in 20 replicates each (see methods) to obtain end structures for electrostatic energy and contact map calculations. (b) Contact maps of monomers, dimers and trimers show extensive contacts between the NTDs and RRMs, and RRMs and LCDs, but not the NTDs and LCDs. (c) Representative example structures of monomers, dimers, and trimers. The NTDs are rendered as surfaces, the RRMs as grey cartoons, and the LCDs as colored cartoons. (d) Electrostatic energy calculations of the monomers, dimers, and trimers at alkaline and neutral pH reveal slightly more favorable energies in the dimers and trimers which is likely due to NTD contacts absent in the monomer. (e) View of the complete TDP-43 oligomer model. The first 8 TDP-43 molecules are shown as full-length, the full NTD "corkscrew" (purple) is shown for 14 molecules.

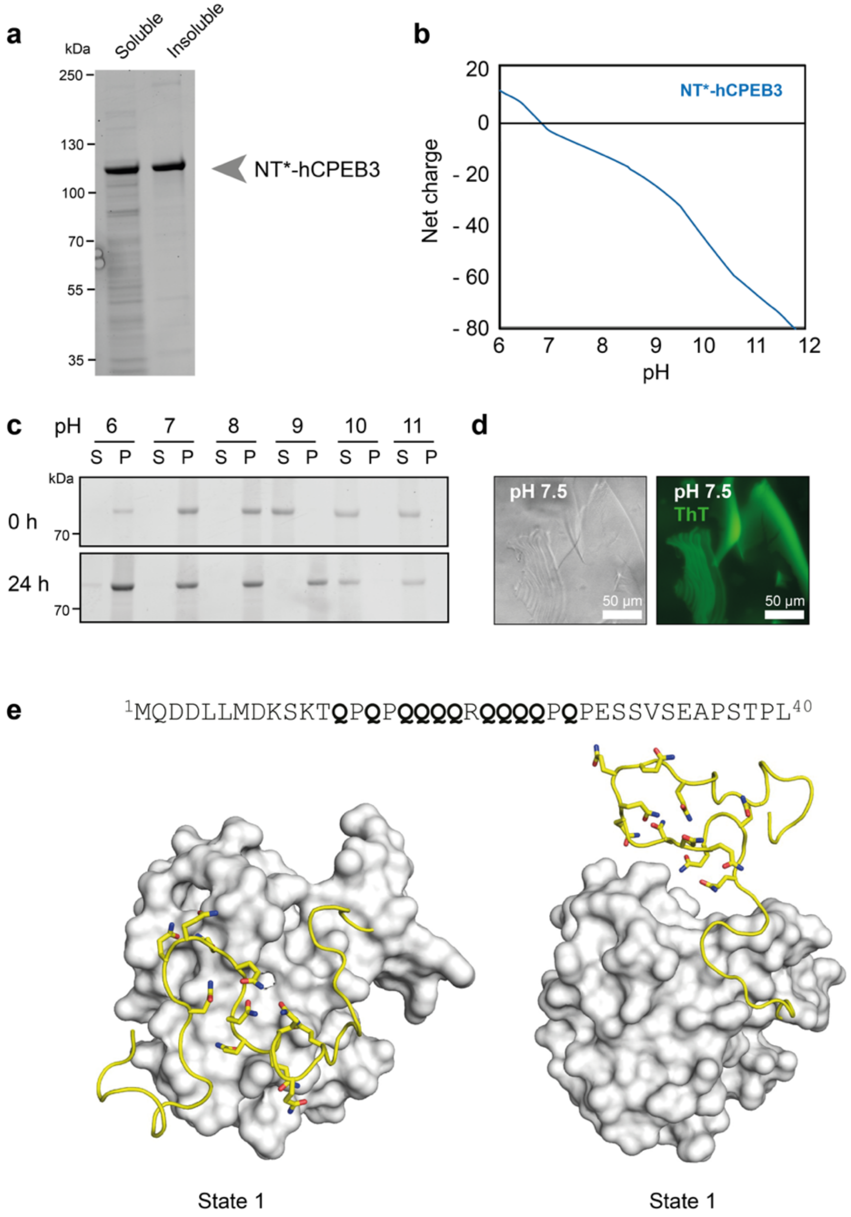

Figure S5. Characterization and interactions in NT*-tagged hCPEB3. (a) Expression of NT*-tagged hCPEB3 yields partially soluble protein as judged by SDS-PAGE. (b) pH-dependent solubility assay shows insoluble NT*-hCPEB3 below pH 8. After 24h, the protein also became insoluble at pH 9. (c) Computed net charge of hCPEB3 from pH 6 to 12. The experimentally observed pH for aggregation is indicated by a shaded area. (d) Fluorescence microscopy of the hCPEB3 assemblies visible by light microscopy shows staining for the amyloid-specific dye ThT, confirming the presence of fibrillar material. (e) MD simulations of NT* fused to the first 40 residues of hCPEB3. The hCPEB3 sequence

is shown above, with the polyglutamine sequence highlighted. Two representative snapshots from the simulations show the polyglutamine stretch bound to the surface of NT* (state 1) or in a flexible conformation facing away from NT* (state 2). The surface of NT* is rendered in grey, the glutamine residues are shown as sticks.

Movie S1.

NT*-tagged FUS diluted from a pH 9 dH2O stock into 20 mM Tris pH 6, 500 mM NaCl, shows phase separation into spherical droplets that fuse on a minute timescale (total 15 minutes). Imaged every 5 sec for 181 frames. Scale bar is 50 μm.
